# Conspiracy beliefs prospectively predict health behavior and well-being during a pandemic

**DOI:** 10.1017/S0033291721004438

**Published:** 2021-10-13

**Authors:** Jan-Willem van Prooijen, Tom W. Etienne, Yordan Kutiyski, André P. M. Krouwel

**Affiliations:** 1 Department of Experimental and Applied Psychology, VU University, Amsterdam; 2 The Netherlands Institute for the Study of Crime and Law Enforcement (NSCR), the Netherlands; 3 Maastricht University, Maastricht, the Netherlands; 4 Kieskompas – Election Compass, Amsterdam, the Netherlands; 5 Department of Political Science, The Pennsylvania State University, PA, USA; 6 Department of Communication Science and Political Science, VU University, Amsterdam

**Keywords:** Compliance, conspiracy theories, Covid-19, health, well-being

## Abstract

**Background:**

Conspiracy beliefs are associated with detrimental health attitudes during the coronavirus disease 2019 (Covid-19) pandemic. Most prior research on these issues was cross-sectional, however, and restricted to attitudes or behavioral intentions. The current research was designed to examine to what extent conspiracy beliefs predict health behavior and well-being over a longer period of time.

**Methods:**

In this preregistered multi-wave study on a large Dutch research panel (weighted to provide nationally representative population estimates), we examined if conspiracy beliefs early in the pandemic (April 2020) would predict a range of concrete health and well-being outcomes eight months later (December 2020; *N* = 5745).

**Results:**

The results revealed that Covid-19 conspiracy beliefs prospectively predicted a decreased likelihood of getting tested for corona; if tested, an increased likelihood of the test coming out positive; and, an increased likelihood of having violated corona regulations, deteriorated economic outcomes (job loss; reduced income), experiences of social rejection, and decreased overall well-being. Most of these effects generalized to a broader susceptibility to conspiracy theories (i.e. conspiracy mentality).

**Conclusions:**

These findings suggest that conspiracy beliefs are associated with a myriad of negative life outcomes in the long run. Conspiracy beliefs predict how well people have coped with the pandemic over a period of eight months, as reflected in their health behavior, and their economic and social well-being.

Since the start of the global coronavirus disease 2019 (Covid-19) pandemic, conspiracy theories about the corona virus have proliferated on the Internet and social media (Loomba, de Figueiredo, Piatek, de Graaf, & Larson, 2021; Pennycook & Rand, 2021). These conspiracy theories have asserted, for instance, that the corona virus is a bioweapon engineered in a Chinese lab, or that the pandemic is a hoax designed by governments to suppress regular citizens (Imhoff & Lamberty, 2020). Conspiracy theories are commonly defined as explanatory beliefs, assuming that a group of actors collude in secret to attain malevolent goals (Bale, 2007). One basic property of conspiracy theories is that they are consequential (Van Prooijen & Douglas, 2018): Even if a conspiracy theory is extremely implausible according to logic or scientific evidence, if it seems real to a perceiver, it has a genuine impact on attitudes, emotions, and behavior (cf. Thomas & Thomas, 1928). Accordingly, experimental studies have revealed that exposure to anti-vaccine conspiracy theories lowers people's favorability toward vaccinating children (Jolley & Douglas, 2014), and that exposure to governmental conspiracy theories increases people's willingness to commit minor forms of crime (e.g. filing false insurance claims; Jolley, Douglas, Leite, & Schrader, 2019). But to what extent do conspiracy beliefs predict important health and well-being outcomes in the long run? The present research sought to answer this question in the context of the Covid-19 pandemic.

Preliminary research has suggested that Covid-19 conspiracy theories are robustly related with a range of perceptions and behavioral intentions that may compromise public health, such as decreased physical distancing, decreased support for restrictive measures, and decreased intentions to get vaccinated (Freeman et al., 2020; Hornsey et al., 2021; Imhoff & Lamberty, 2020; Marinthe, Brown, Delouvée, & Jolley, 2020). These findings underscore the relevance of conspiracy theories to understand human responses to the pandemic. Most of these findings are cross-sectional however, although some evidence reveals that conspiracy beliefs predict a progressive decrease in physical distancing in a longitudinal design, over a relatively short time span (from March to April 2020; Bierwiaczonek, Kunst, & Pich, 2020). Furthermore, most health-relevant responses were in previous research necessarily measured as attitudes or behavioral intentions. It is therefore yet unclear whether Covid-19 conspiracy theories predict concrete behavioral outcomes in the context of health or well-being. More generally, prospective or longitudinal studies are scarce in the scientific study of conspiracy theories (for exceptions, see Bierwiaczonek *et al*., 2020; Golec de Zavala & Federico, 2018), and little is known about the prospective predictive power of conspiracy beliefs over longer periods of time (Van Prooijen & Douglas, 2018).

This research study aims to make a novel contribution by investigating how conspiracy beliefs predict a range of concrete health and well-being outcomes over time during the Covid-19 pandemic. On a large panel, weighted to provide nationally representative estimates for the Dutch adult population, we solicited multiple waves during the course of the pandemic in 2020. The study first measured conspiracy beliefs early in the pandemic. Then, in a preregistered follow-up wave we assessed how well conspiracy beliefs would predict a range of concrete and binary outcomes eight months later, in the context of health (e.g. Did participants get tested for corona; and if so, was the test positive? Have they violated specific restrictive measures to contain the spread of the virus?), and well-being (e.g. Have participants suffered a loss of income? Did they experience social rejection?). In the following section, we introduce our line of reasoning and hypotheses.

## The current research

While conspiracy theories may differ widely in content, belief in such theories is grounded in similar and predictable psychological processes (e.g. Butter & Knight, 2020; Douglas, Cichocka, & Sutton, 2017; Uscinski & Parent, 2014; Van Prooijen, 2018, 2020; Van Prooijen & Van Vugt, 2018). As such, belief in one conspiracy theory is a good predictor of belief in a different conspiracy theory, suggesting that people structurally differ in their susceptibility to conspiracy theories (Goertzel, 1994; Swami et al., 2011; Wood, Douglas, & Sutton, 2012). Indeed, scholars have frequently investigated conspiracy thinking by operationalizing conspiracy mentality—a dispositional tendency to explain events in the world with the belief that they are caused by harmful conspiracies (Bruder, Haffke, Neave, Nouripanah, & Imhoff, 2013; Imhoff & Bruder, 2014). Notwithstanding the notion that situational factors (e.g. social crisis situations; intergroup conflict; culture) also significantly contribute to conspiracy thinking (Crocker, Luhtanen, Broadnax, & Blaine, 1999; Van Prooijen & Douglas, 2017; Van Prooijen & Song, 2021; Whitson & Galinsky, 2008), their trait-like qualities suggest that conspiracy beliefs at one point in time should be a good predictor of conspiracy beliefs at a later point in time.

Combining these arguments with research findings suggesting a link between conspiracy beliefs and a range of perceptions and behavioral intentions that undermine personal and public health, we propose that conspiracy beliefs early in the pandemic prospectively predict a range of health-related behavioral outcomes months later. Specifically, as conspiracy thinking is associated with feeling less threatened by the corona virus and reduced willingness to follow containment guidelines (Freeman et al., 2020), we expected that conspiracy beliefs would predict a decreased likelihood of getting tested for corona (Hypothesis 1). These arguments also imply that conspiracy thinking (a) makes asymptomatic testing less likely and (b) puts people at greater risk for infection. Combining these arguments, we expected that among those who did get tested, conspiracy beliefs would predict an increased likelihood of the test coming out positive (Hypothesis 2).

Furthermore, the study examined whether conspiracy beliefs would predict compliance with restrictive regulations designed to protect public health. Building on prior research showing a relationship between conspiracy beliefs and decreased support for government-imposed restrictive measures (Freeman et al., 2020; Hornsey et al., 2021; Imhoff & Lamberty, 2020; Marinthe et al., 2020), we hypothesized that conspiracy beliefs would predict increased likelihoods of receiving a fine for violating the corona regulations (Hypothesis 3), of receiving more visitors in one's home, and visiting a party or bar/restaurant that is more crowded than allowed (Hypothesis 4). Moreover, the study examined the prospective effects of conspiracy beliefs on face mask wearing. Particularly in the Netherlands, face masks were highly unusual among the general public early in the pandemic (i.e. initially, both the Dutch government and health authorities proclaimed that face masks were ineffective; moreover, there was a major shortage of them). Only months later face masks grew more customary, and eventually became mandatory in indoor public places. As such, showing prospective effects of conspiracy beliefs on face mask wearing provides a relatively strong test of our line of reasoning. We hypothesized that conspiracy thinking in April 2020 would predict a decreased likelihood of wearing face masks in December 2020 (Hypothesis 5).

Besides the implications for (personal and public) health, the study also examined if conspiracy beliefs would prospectively predict other aspects of people's well-being. We propose that conspiracy beliefs are associated with decreased social resources necessary to cope with the pandemic. Specifically, conspiracy beliefs carry a social stigma, and publicly spreading conspiracy theories therefore makes people prone to social exclusion (Lantian et al., 2018). Moreover, Covid-19 conspiracy beliefs are associated with self-centeredness, as for instance reflected in hoarding behavior or a decreased concern for other's safety (Hornsey et al., 2021; Imhoff & Lamberty, 2020), which may deteriorate social relationships. Conspiracy beliefs hence are likely to be associated with decreased levels of social support, which has numerous implications for well-being. In particular, we expected that conspiracy beliefs early in the pandemic would predict increased economic problems later in the pandemic (Hypothesis 6), operationalized as job loss and reduced income. Moreover, we hypothesized that conspiracy beliefs would predict deteriorated social relationships (Hypothesis 7) and a more generally decreased well-being (Hypothesis 8).

## Open practices statement

The hypotheses were preregistered on the Open Science Framework (OSF) prior to running the final wave containing the dependent variables. An anonymized copy of the data, the analysis code necessary to reproduce the results, and other materials can be found on OSF.^†^^1^

## Method

### Data collection and sample

The study was part of a large-scale, three-wave measurement project on a large Dutch population panel. The data were collected by Kieskompas (‘Election compass’), a political research institute that specializes in panel research. Kieskompas fully adheres to GDPR (i.e. EU privacy) regulations, is closely monitored by the Dutch privacy authority, and acts in line with the ethical norms of VU Amsterdam. The panels were acquired through Voting Advice Applications prior to elections as well as through targeted survey studies to complement the sampling framework, and participants received an email invitation and reminder to participate in each wave. The study was part of a large research project on the psychological, moral, and political dynamics that are relevant to understand human behavior during the Covid-19 pandemic (Krouwel, Etienne, & Kutiyski, 2020).

As preregistered, for the present purposes we have only used measurements from Wave 1 (April 2020; from now on referred to as ‘T1’) and Wave 3 (December 2020; from now on referred to as ‘T2’). These measurement points were drawn during the first (T1) and second (T2) peak of infections (both including strict lockdown measures) in the Netherlands. Wave 1 had a total of 9033 participants; of these, 5745 respondents also participated in the last wave (63.60%; predictors of attrition analyzed in the Supplementary Materials on OSF). This sample forms the basis of the present analyses (raw unweighted demographics, 4070 men, 1675 women; *M*_age_ = 58.74, CI_95%_ (58.34–59.13); actual sample varies between dependent measures due to missing values). The data of the last wave were weighted in the analyses to provide nationally representative population estimates, through poststratification and iterative proportional fitting with benchmarks age, sex, education, geographical region, ethnicity, and vote recall, relying on the Dutch golden standard (CBS) as well as the official 2017 parliamentary election results (for more details about weighting in non-probability samples, see Baker et al., 2013). All participants gave their informed consent.

### Procedure

The first wave took place early in the pandemic in the Netherlands (April 2020). This wave assessed participants' gender, age, education level (1 = *university master*, 7 = *no education / primary school*; recoded), and their self-reported political ideology on a slider (0 = *very left-wing*, 10 = *very right-wing*). Furthermore, it contained a four-item measure of Covid-19 conspiracy beliefs (Van Bavel et al., 2021) measured on a slider ranging from 0 (*strongly disagree*) to 10 (*strongly agree*): ‘The coronavirus (COVID-19) is a bioweapon engineered by scientists’, ‘The coronavirus (COVID-19) is a conspiracy to take away citizens’ rights for good and establish an authoritarian government’, ‘The coronavirus (COVID-19) is a hoax invented by interest groups for financial gains’, and ‘The coronavirus (COVID-19) was created as a cover-up for the impending global economic crash’. The scale had good reliability (*α* = 0.91), although on average, Covid-19 conspiracy belief was low in the sample (*M* = 1.03), CI_95%_ (0.98–1.08).^2^

Furthermore, the first wave at T1 contained the 5-item conspiracy mentality scale (Bruder et al., 2013), which measures participants' general predisposition to believe conspiracy theories (*α* = 0.88; example item: ‘I think that many very important things happen in the world, which the public is never informed about’; 1 = *0% certainly not*, to 11 = *100% certainly*). Participants displayed moderate levels of conspiracy mentality (*M* = 4.92), CI_95%_ (4.86–4.98).

The final wave at T2 (December 2020) included a range of concrete behavioral outcomes, measured in a binary format (1 = *No*, 2 = *Yes*). Two of these outcomes referred to *testing*: ‘Since the beginning of the pandemic, did you get tested (either once or multiple times) for corona?’, and ‘Did you test positively for the coronavirus (COVID-19), meaning that you (now or earlier) have received a medical confirmation of this disease?’. Other outcomes referred to *compliance with corona regulations*: ‘Since the beginning of the pandemic, did you (either once or multiple times) get a fine for violating the corona regulations?’, ‘Since the beginning of the pandemic, did you (either once or multiple times) receive more visitors in your house than allowed at that time?’, and ‘Since the beginning of the pandemic, did you (either once or multiple times) visit a party or bar / restaurant where it was more crowded than allowed at that time?’. Two items referred to *economic outcomes*: ‘Did you lose your job during the pandemic?’, and ‘Did you experience a reduced income during the pandemic?’. Finally, two items referred to *social relationships*: ‘Because of my opinion on corona, some people do not want to have contact with me anymore’, and ‘I terminated contact with people due to the things they say about corona’.

Besides these dichotomous behavioral outcomes, the questionnaire also included various Likert scales to further assess some of the constructs of interest. As to compliance, we also assessed participants' tendency to wear face masks. In the Netherlands, since 1 June 2020 face masks became recommended, and mandatory in public transport; shortly before implementing the final wave (1 December 2020), they also became mandatory in indoor public spaces. We assessed the following two items on face mask wearing (1 = *Never,* 5 = *Always*): ‘How often do you currently wear a face mask in public places?’, and ‘How often did you wear a face mask *before* it became mandatory on 1 December?’. These items were strongly correlated (*r* = 0.47, *p* < 0.001) and we averaged them into a single indicator of face mask wearing (*M* = 4.02), CI_95%_ (3.99–4.04).

To further assess participants' economic outcomes, the questionnaire included the following question: ‘How did the COVID-19 pandemic influence the financial situation of your household?’ (1 = *Made it much worse*, 5 = *Made it much better*) (an additional response option, ‘*not applicable / don't know*’ was coded as missing) (*M* = 2.84), CI_95%_ (2.82–2.86).

Finally, the questionnaire included a measure of how participants' well-being has been harmed due to the pandemic. Participants were specifically asked how often they have experienced the following problems during the pandemic, as compared to before the pandemic (1 = *much less than before the pandemic*, 5 *=* *much more than before the pandemic*): Financial problems; relationship problems; loneliness; depression; fear; uncertainty about the future; conflicts; sleeplessness; frustration; temper tantrums; panic attacks. These items were averaged into a single indicator of participants' experienced personal problems (*α* = 0.87; *M* = 3.18), CI_95%_ (3.16–3.19).

## Results

We first conducted a confirmatory test of our hypotheses, pertaining to Covid-19 conspiracy beliefs. As preregistered, in all our analyses we controlled for gender, age, political orientation, and education level in Step 1 of our logistic or linear regression models (all tests two-sided). After that, we exploratively conducted the same analyses for conspiracy mentality, for two reasons: First, such an analysis provides an extra validation that is useful given the relatively low levels of specific Covid-19 conspiracy beliefs in the sample; and second, it enables us to establish if the observed effects generalize beyond specific Covid-19 conspiracy beliefs, to a dispositional tendency to be susceptible to conspiracy theories.

**Fig. 1.**
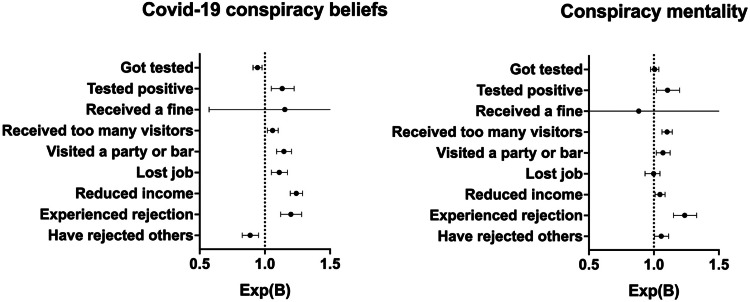
Odds ratios Exp(B) and 95% confidence intervals of health behavior and well-being as a function of Covid-19 conspiracy beliefs and conspiracy mentality.

In line with our preregistered protocol to focus on nationally representative population estimates, the results reported below are for the weighted sample. Some of the results were different for the unweighted sample, however^3^; for full disclosure, we therefore provide a full overview of both the weighted and unweighted results in online Supplementary Table S1 (provided on OSF). Moreover, for an efficient presentation of the results, we also refer to online Supplementary Table S1 for a complete overview of the effects of the control variables. The odds ratios for the various binary health and well-being outcomes as a function of Covid-19 conspiracy beliefs and conspiracy mentality are displayed in Fig. 1.

## Confirmatory analyses: covid-19 conspiracy beliefs

The results of the hierarchical logistic regression analyses are displayed in Table 1.

**Table 1.** Hierarchical logistic regression results: Covid-19 conspiracy beliefs as predictor of binary health and well-being outcomes eight months later

	*χ*^2^(df)	Nagelkerke *R*^2^	*B*(se)	Wald	Exp(*B*)	CI_95%_
Got tested						
Step 1	162.76 (4)***	0.046				
Step 2	10.38 (1)**	0.049	−0.059 (0.019)**	10.12	0.943	0.909–0.978
Tested positive						
Step 1	90.63 (4)***	0.089				
Step 2	9.42 (1)**	0.098	0.125 (0.039)**	10.02	1.133	1.049–1.224
Received a fine						
Step 1	4.71 (4)	0.126				
Step 2	0.12 (1)	0.130	0.142 (0.357)	0.16	1.153	0.572–2.323
Received too many visitors						
Step 1	497.55 (4)***	0.148				
Step 2	8.20 (1)**	0.151	0.058 (0.020)**	8.36	1.059	1.019–1.102
Visited a party or bar						
Step 1	537.45 (4)***	0.205				
Step 2	27.26 (1)***	0.215	0.136 (0.025)***	28.86	1.146	1.090–1.204
Lost job						
Step 1	255.44 (4)***	0.117				
Step 2	12.96 (1)***	0.123	0.104 (0.028)***	13.78	1.110	1.050–1.173
Reduced income						
Step 1	71.48 (4)***	0.024				
Step 2	122.24 (1)***	0.064	0.216 (0.019)***	125.96	1.241	1.195–1.289
Experienced rejection						
Step 1	47.98 (4)***	0.035				
Step 2	24.62 (1)***	0.052	0.182 (0.034)***	28.07	1.199	1.121–1.282
Have rejected others						
Step 1	28.38 (4)***	0.014				
Step 2	12.66 (1)***	0.019	−0.120 (0.036)***	11.14	0.887	0.826–0.952

*Note.* Step 1 contains the control variables gender, age, political orientation, and education (details about their specific effects in online Supplementary Table S1). Step 2 adds Covid-19 conspiracy beliefs to the regression model. * *p* < 0.05; ** *p* < 0.01; *** *p* < 0.001.

### Covid-19 testing

The results supported Hypotheses 1 and 2. Believing in Covid-19 conspiracy beliefs at T1 (April 2020) predicted a decreased likelihood of having been tested by T2 (December 2020). Among those who did get tested, conspiracy beliefs also predicted an increased likelihood of the test coming out positive.

### Compliance with corona regulations

Hypotheses 3−5 all pertained to compliance with the corona regulations. We first assessed if participants had received a fine for violating the corona regulations. Only two participants in our sample had received a fine, however, and both steps of the logistic regression model were indeed nonsignificant (*p*s > 0.31). Hypothesis 3 was hence not supported.

The results did support Hypothesis 4, on both indicators (see Table 1). Covid-19 conspiracy beliefs at T1 predicted an increased likelihood of having received too many visitors in one's home by T2. Also, conspiracy beliefs at T1 predicted an increased likelihood of having visited an overcrowded party or bar/restaurant at T2.

As preregistered, we used hierarchical linear regression analysis to analyze the scale of face mask wearing. Step 1 including the control variables was significant (*R*^2^ = 0.027), *F*(4, 4728) = 33.085, *p* < 0.001. More important for the present purposes, Step 2 was significant, (Δ*R*^2^ = 0.072), *F*(1, 4727) = 375.305, *p* < 0.001. Supporting Hypothesis 5, conspiracy beliefs at T1 predicted decreased face mask wearing at T2, *B* = −0.142, se = 0.007; CI_95%_ (−0.156 to −0.127), *p* < 0.001.

### Economic outcomes

A total of 332 participants indicated to have lost their job during the pandemic. As predicted, hierarchical logistic regression results revealed that conspiracy beliefs at T1 predicted an increased likelihood of job loss, and a loss of income, at T2 (see Table 1). We also analyzed the Likert-scale question measuring to what extent the pandemic has negatively or positively influenced the finances of participants' household through a hierarchical linear regression. Step 1 was significant (*R*^2^ = 0.020), *F*(4, 4546) = 23.452, *p* < 0.001. Mirroring the findings on the binary question about loss of income, Step 2 was also significant (Δ*R*^2^ = 0.026), *F*(1, 4545) = 123.248, *p* < 0.001, indicating that conspiracy beliefs at T1 predicted worse finances at T2, *B* = −0.068, se = 0.006; CI_95%_ (−0.080 to −0.056), *p* < 0.001. In sum, the results supported Hypothesis 6, that conspiracy beliefs in April 2020 predict increased economic problems in December 2020.

### Social relationships

We then examined Hypothesis 7 that conspiracy beliefs at T1 predicts disrupted social relationships at T2. On the question whether other people have ended social contacts with the participant due to their opinions on corona, logistic regression results revealed that conspiracy beliefs at T1 predicted an increased likelihood that others have ended contact with the participant at T2. With regard to the question whether the participant has ended contact with other people due to what they say about corona, however, results showed an effect opposite to predictions: Particularly people *low* on conspiracy thinking were likely to end contact with others due to what they say about corona (see Table 1).

These results provide mixed support for Hypothesis 7 that conspiracy beliefs in April 2020 predict an increased chance of disrupted social relationships by December 2020. Instead, these findings suggest that it is more common for people high on conspiracy beliefs to experience social rejection. Presumably, people low in conspiracy belief are more likely to reject people high in conspiracy belief rather than vice versa. Such intolerance of conspiracy believers is consistent with the notion that publicly endorsing conspiracy beliefs is stigmatizing and can decrease people's social support network (Lantian et al., 2018; see also Hornsey et al., 2021; Imhoff & Lamberty, 2020; Van Prooijen, Spadaro, & Wang, 2022).

### Experienced problems

To test Hypothesis 8 that conspiracy beliefs at T1 predict decreased well-being at T2, we analyzed the scale of experienced personal problems during the pandemic. Step 1 was significant (*R*^2^ = 0.048), *F*(4, 4729) = 60.153, *p* < 0.001. More importantly, Step 2 was significant (Δ*R*^2^ = 0.005), *F*(1, 4728) = 26.374, *p* < 0.001. As predicted, conspiracy beliefs at T1 were associated with increased experienced problems – and hence decreased well-being – at T2, *B* = 0.015, se = 0.003; CI_95%_ (0.010–0.021), *p* < 0.001.

## Exploratory analyses: conspiracy mentality

We then repeated all these analyses for conspiracy mentality at T1, and report the results for the hierarchical logistic regression analyses in Table 2. Conspiracy mentality did not predict an increased likelihood of getting tested eight months later; among those that did get tested, however, conspiracy mentality did predict an increased likelihood of the test being positive.

**Table 2.** Hierarchical logistic regression results: conspiracy mentality as predictor of binary health and well-being outcomes eight months later

	*χ*^2^(df)	Nagelkerke *R*^2^	*B*(se)	Wald	Exp(*B*)	CI_95%_
Got tested						
Step 1	155.22(4)***	0.047				
Step 2	0.15(1)	0.005	0.006(0.016)	0.15	1.006	0.975–1.038
Tested positive						
Step 1	98.11(4)***	0.101				
Step 2	6.01(1)*	0.107	0.100(0.041)*	6.01	1.105	1.020–1.198
Received a fine						
Step 1	4.65(4)	0.126				
Step 2	0.10(1)	0.128	−0.124(0.395)	0.10	0.883	0.408–1.915
Received too many visitors						
Step 1	457.35(4)***	0.145				
Step 2	27.99(1)***	0.153	0.096(0.018)***	27.97	1.101	1.062–1.141
Visited a party or bar						
Step 1	605.48(4)***	0.247				
Step 2	7.10(1)**	0.249	0.068(0.026)**	7.13	1.070	1.018–1.125
Lost job						
Step 1	230.71(4)***	0.123				
Step 2	0.17(1)	0.123	−0.012(0.030)	0.17	0.998	0.932–1.047
Reduced income						
Step 1	75.00(4)***	0.027				
Step 2	5.86(1)*	0.029	0.046(0.019)*	5.88	1.047	1.009–1.086
Experienced rejection						
Step 1	43.87(4)***	0.033				
Step 2	33.26(1)***	0.058	0.212(0.037)***	33.24	1.237	1.150–1.329
Have rejected others						
Step 1	22.23(4)***	0.011				
Step 2	4.37(1)*	0.013	0.056(0.027)*	4.39	1.057	1.004–1.114

*Note.* Step 1 contains the control variables gender, age, political orientation, and education (details about their specific effects in online Supplementary Table S1). Step 2 adds conspiracy mentality to the regression model. * *p* < 0.05; ** *p* < 0.01; *** *p* < 0.001.

As might be expected given the low number of people that received a fine, conspiracy mentality was unrelated with the likelihood of a fine for violating the corona regulations. Conspiracy mentality did predict an increased likelihood of receiving too many visitors in one's home and attending an overcrowded party or bar/restaurant, however (Table 2). Also, increased conspiracy mentality in April 2020 was associated with decreased mouth-mask wearing in December 2020 (Δ*R*^2^ = 0.046), *F*(1, 4443) = 223.246, *p* < 0.001; *B* = −0.099, se = 0.007; CI_95%_ (−0.112 to −0.086), *p* < 0.001.

Unlike the findings for Covid-19 conspiracy beliefs, conspiracy mentality did not predict an increased chance of losing one's job. Conspiracy mentality did significantly predict a loss of income, however, both on the dichotomous indicator (Table 2), and the continuous indicator (Δ*R*^2^ = 0.003), *F*(1, 4287) = 12.619, *p* < 0.001; *B* = −0.020, se = 0.005; CI_95%_ (−0.030 to −0.009), *p* < 0.001.

As also observed for Covid-19 conspiracy beliefs, conspiracy mentality predicted an increased likelihood that other people had terminated contact with the participant due to their opinions on corona. Unlike the findings for Covid-19 conspiracy beliefs, however, conspiracy mentality predicted an increased likelihood of also terminating contact with other people, although the effect size was small (see Table 2). Finally, conspiracy mentality predicted increased problems due to the pandemic (Δ*R*^2^ = 0.002), *F*(1, 4445) = 10.657, *p* = 0.001; *B* = 0.009, se = 0.003; CI_95%_ (0.004–0.014), *p* = 0.001.

In sum, the general conspiracy mentality trait prospectively predicts health and well-being outcomes in a largely comparable manner as specific Covid-19 conspiracy beliefs, with the exceptions of getting tested and job loss (which were both nonsignificant for conspiracy mentality), and terminating contact with other people (which was negative for Covid-19 conspiracy beliefs but positive for conspiracy mentality).

## Discussion

The present study suggests that believing conspiracy theories early in the pandemic predicts a range of health and well-being outcomes eight months later. Specifically, endorsing Covid-19 conspiracy theories in April 2020 predicts whether by December 2020 participants have been tested for corona, whether that test came out positive, whether they have violated regulations to contain the spread of the corona virus, whether they suffer from economic problems (in the form of job loss and reduced income), whether they have experienced rejection in their social relationships, and whether their well-being has deteriorated. Most of these effects also generalize to a dispositional tendency to believe conspiracy theories (i.e. conspiracy mentality). These findings underscore that conspiracy thinking is relevant for people's health and well-being over time.

The present findings meaningfully extend previous research on the link between conspiracy theories and health-related responses in the context of the Covid-19 pandemic, in at least two ways. First, most prior research on this link has been cross-sectional, raising alternative explanations that are conceptual (e.g. conspiracy theories as a mental tool to justify opposition against the restrictive measures; Mercier, 2020), and methodological (e.g. common method variance; Podsakoff, MacKenzie, Lee, & Podsakoff, 2003). The present findings provide more solid evidence for these links by showing that conspiracy beliefs have meaningful implications for health and well-being eight months later. Second, previous research has predominantly examined attitudes and behavioral intentions that are relevant during the pandemic, not actual behavioral outcomes. While also in the current study it was impossible to directly observe actual behavior, many of the behavioral or life outcomes assessed here are concrete, do not require intensive memory reconstructions, and can be answered in a binary format (e.g. Did participants get tested for corona?). As such, the present findings provide relatively direct evidence for a link between conspiracy beliefs and actual behavioral or life outcomes during the Covid-19 pandemic.

Although the current study has a number of strengths (e.g. the large sample with nationally representative population estimates, and the multi-wave design), there are also a number of limitations. First, Covid-19 conspiracy beliefs were low in the sample. Other studies also suggest that while many people endorse general Covid-19 conspiracy theories (e.g. a general sentiment that authorities hide the truth about the pandemic), endorsement of more specific Covid-19 conspiracy theories – such as the belief that the virus is a bioweapon – is much lower (Hornsey et al., 2021). For the present purposes, we note that most of the results replicated for the general trait conspiracy mentality, supporting the validity of our conclusions. Yet, this issue does suggest that follow-up studies may include more general Covid-19 conspiracy theories than assessed here.

As a second limitation, although our findings are consistent with a theoretical argument that conspiracy beliefs causally shape health and well-being outcomes over time, our design does not allow firm conclusions about causality. After all, our design precluded the possibility to control for the autocorrelations from T1 to T2 for the dependent measures. Moreover, for some of the dependent measures more specific alternative explanations exist. For instance, it is possible that people high on conspiracy beliefs more selectively remember (or, as act of protest, more proudly report) instances where they violated corona regulations. Likewise, people with professions vulnerable to lockdown measures (e.g. shop or bar owners) may have been particularly likely to develop conspiracy theories, providing an alternative explanation for the link with economic outcomes. Finally, it is possible that conspiracy beliefs are associated with a generally pessimistic outlook on one's life and the world, prompting relatively negative appraisals of how the pandemic has affected one's well-being. These issues suggest that more research is required to disentangle these complex relationships, and establish the long-term causal effects of conspiracy thinking on health and well-being.

To conclude, conspiracy theories have received a lot of attention during the pandemic, on the Internet, social media, and public discourse. While both theorizing and empirical research have suggested that conspiracy beliefs may predict health and well-being, thus far these associations have only been established in the short run. The present findings add to this body of research by establishing the role of conspiracy theories in the long run. Conspiracy beliefs predict how well people cope with the challenges of a global pandemic, and therefore has substantial implications for private and public health, as well as perceivers' economic and social well-being.

## Footnotes

† The notes appear after the main text.

## Notes

1 Preregistration: https://osf.io/e2pk3. Data, code, supplementary materials, and online Supplementary Table S1: https://osf.io/cmd8g/.

2 The same scale was also assessed at T2. The correlation of Covid-19 conspiracy beliefs between T1 and T2 was substantial (*r* = 0.71, *p* <0.001), confirming our assumption that such beliefs are relatively stable over time.

3 Specifically, in the unweighted sample, Hypothesis 2 was not supported, and Hypothesis 6 was supported only for reduced income, not for job loss.

## Supplementary material

For supplementary material accompanying this paper visit http://dx.doi.org/10.1017/S0033291721004438. click here to view supplementary material

## References

[ref1] Baker, R., Brick, J. M., Bates, N. A., Battaglia, M., Couper, M. P., Dever, J. A., … Tourangeau, R. (2013). Summary report of the AAPOR task force on Non-probability sampling. Journal of Survey Statistics and Methodology, 1(2), 90–143.

[ref2] Bale, J. M. (2007). Political paranoia v. Political realism: On distinguishing between bogus conspiracy theories and genuine conspiratorial politics. Patterns of Prejudice, 41, 45–60.

[ref3] Bierwiaczonek, K., Kunst, J. R., & Pich, O. (2020). Belief in COVID-19 conspiracy theories reduces social distancing over time. Applied Psychology: Health and Well-Being, 12, 1270–1285. 32864837 10.1111/aphw.12223

[ref4] Bruder, M., Haffke, P., Neave, N., Nouripanah, N., & Imhoff, R. (2013). Measuring individual differences in generic belief in conspiracy theories across cultures: Conspiracy mentality questionnaire. Frontiers in Psychology, 4:225. 23641227 10.3389/fpsyg.2013.00225 PMC3639408

[ref5] Butter, M., & Knight, P. (2020). Routledge handbook of conspiracy theories. Oxon, UK: Routledge.

[ref6] Crocker, J., Luhtanen, R., Broadnax, S., & Blaine, B. E. (1999). Belief in U.S. Government conspiracies against blacks among black and white college students: Powerlessness or system blame? Personality and Social Psychology Bulletin, 25, 941–953.

[ref7] Douglas, K. M., Cichocka, A., & Sutton, R. M. (2017). The psychology of conspiracy theories. Current Directions in Psychological Science, 26, 538–542. 29276345 10.1177/0963721417718261 PMC5724570

[ref8] Freeman, D., Waite, F., Rosebrock, L., Petit, A., Causier, C., East, A., … Lambe, S. (2020). Coronavirus conspiracy beliefs, mistrust, and compliance with government guidelines in England. Psychological Medicine, 1–13. 10.1017/S0033291720001890. PMC7264452 32436485

[ref9] Goertzel, T. (1994). Belief in conspiracy theories. Political Psychology, 15, 733–744.

[ref10] Golec de Zavala, A., & Federico, C. M. (2018). Collective narcissism and the growth of conspiracy thinking over the course of the 2016 United States presidential election: A longitudinal analysis. European Journal of Social Psychology, 48, 1011–1018.

[ref11] Hornsey, M. J., Chapman, C. M., Alvarez, B., Bentley, S., Casara, B. G. S., Crimston, C. R., … Jetten, J. (2021). To what extent are conspiracy theorists concerned for self versus others? A COVID-19 test case. European Journal of Social Psychology, 51, 285–293. doi:10.1002/EJSP.2737. PMC8014880 33821057

[ref12] Imhoff, R., & Bruder, M. (2014). Speaking (un-)truth to power: Conspiracy mentality as a generalized political attitude. European Journal of Personality, 28, 25–43.

[ref13] Imhoff, R., & Lamberty, P. (2020). A bioweapon or a hoax? The link between distinct conspiracy beliefs about the coronavirus disease (COVID-19) outbreak and pandemic behavior. Social Psychological and Personality Science, 11, 1110–1118. 10.1177/1948550620934692 PMC7342934 38602949

[ref14] Jolley, D., & Douglas, K. (2014). The effects of anti-vaccine conspiracy theories on vaccination intentions. PLoS ONE, 9, e89177. 24586574 10.1371/journal.pone.0089177 PMC3930676

[ref15] Jolley, D., Douglas, K. M., Leite, A. C., & Schrader, T. (2019). Belief in conspiracy theories and intentions to engage in everyday crime. British Journal of Psychology, 58, 534–549. 10.1111/bjso.12311 30659628

[ref16] Krouwel, A., Etienne, T., & Kutiyski, Y. (2020). Kieskompas survey data: Public opinion in the time of COVID-19. Amsterdam, the Netherlands: Kieskompas.

[ref17] Lantian, A., Muller, D., Nurra, C., Klein, O., Berjot, S., & Pantazi, M. (2018). Stigmatized beliefs: Conspiracy theories, anticipated negative evaluation of the self, and fear of social exclusion. European Journal of Social Psychology, 48, 939–954.

[ref18] Loomba, S., de Figueiredo, A., Piatek, S. J., de Graaf, K., & Larson, H. J. (2021). Measuring the impact of COVID-19 vaccine misinformation on vaccination intent in the UK and USA. Nature Human Behaviour, 5, 337–348. . 10.1038/s41562-021-01056-1 33547453

[ref19] Marinthe, G., Brown, G., Delouvée, S., & Jolley, D. (2020). Looking out for myself: Exploring the relationship between conspiracy mentality, perceived personal risk, and COVID-19 prevention measures. British Journal of Health Psychology, 25, 957–980. 32583540 10.1111/bjhp.12449 PMC7361332

[ref20] Mercier, H. (2020). Not born yesterday: The science of who we trust and what we believe. Princeton, NJ: Princeton University Press.

[ref21] Pennycook, G., & Rand, D. G. (2021). The psychology of fake news. Trends in Cognitive Sciences, 25, 388–402. 33736957 10.1016/j.tics.2021.02.007

[ref22] Podsakoff, P. M., MacKenzie, S. B., Lee, J.-Y., & Podsakoff, N. P. (2003). Common method bias in behavioral research: A critical review of the literature and recommended remedies. Journal of Applied Psychology, 88, 879–903. 10.1037/0021-9010.88.5.879 14516251

[ref23] Swami, V., Coles, R., Stieger, S., Pietschnig, J., Furnham, A., Rehim, S., & Voracek, M. (2011). Conspiracist ideation in Britain and Austria: Evidence of a monological belief system and associations between individual psychological differences and real-world and fictitious conspiracy theories. British Journal of Psychology, 102, 443–463. 21751999 10.1111/j.2044-8295.2010.02004.x

[ref24] Thomas, W. I., & Thomas, D. S. (1928). The child in America: Behavior problems and programs. New York, NY: Knopf.

[ref25] Uscinski, J. E., & Parent, J. M. (2014). American conspiracy theories. New York, NY: Oxford University Press.

[ref26] Van Bavel, J. J., Cichocka, A., Capraro, V., Sjåstad, H., Nezlek, J. B., Pavlovic, T., Alfano, M, … Boggio, P. S. (2021). National identity predicts public health support during a global pandemic: Results from 67 nations. PsyArXiv preprint. 10.31234/osf.io/ydt95.

[ref27] Van Prooijen, J.-W. (2018). The psychology of conspiracy theories. Oxon, UK: Routledge.

[ref28] Van Prooijen, J.-W. (2020). An existential threat model of conspiracy theories. European Psychologist, 25, 16–25.

[ref29] Van Prooijen, J.-W., & Douglas, K. M. (2017). Conspiracy theories as part of history: The role of societal crisis situations. Memory Studies, 10, 323–333. 29081831 10.1177/1750698017701615 PMC5646574

[ref30] Van Prooijen, J.-W., & Douglas, K. M. (2018). Belief in conspiracy theories: Basic principles of an emerging research domain. European Journal of Social Psychology, 48, 897–908. 30555188 10.1002/ejsp.2530 PMC6282974

[ref31] Van Prooijen, J.-W., & Song, M. (2021). The cultural dimension of intergroup conspiracy theories. British Journal of Psychology, 112, 455–473. 10.1111/bjop.12471. 32790180 PMC8246844

[ref32] Van Prooijen, J.-W., Spadaro, G., & Wang, H. (2022). Suspicion of institutions: How distrust and conspiracy theories deteriorate social relationships. Current Opinion in Psychology, 43, 65–69. 10.1016/j.copsyc.2021.06.013 34298201

[ref33] Van Prooijen, J.-W., & Van Vugt, M. (2018). Conspiracy theories: Evolved functions and psychological mechanisms. Perspectives on Psychological Science, 13, 770–788. 30231213 10.1177/1745691618774270 PMC6238178

[ref34] Whitson, J. A., & Galinsky, A. D. (2008). Lacking control increases illusory pattern perception. Science (New York, N.Y.), 322, 115–117. 10.1126/science.1159845 18832647

[ref35] Wood, M. J., Douglas, K. M., & Sutton, R. M. (2012). Dead and alive: Beliefs in contradictory conspiracy theories. Social Psychological and Personality Science, 3, 767–773.

