# Supplementary material for: Conspiracy beliefs prospectively predict health behavior and well-being during a pandemic
Source: Psychol Med. 2021 Oct 13;53(6):2514–21. doi: 10.1017/S0033291721004438 (PMC8529348; doi:10.1017/S0033291721004438)
Supplement: Supplementary file 1 [file S0033291721004438sup001.docx]

**Conspiracy Beliefs Prospectively Predict Health Behavior and Well-being during a Pandemic**

Supplementary Materials

Jan-Willem van Prooijen^1,2,3^, Tom W. Etienne^4,5^, Yordan Kutiyski^4^, & André P. M. Krouwel^6^

^1^ VU Amsterdam, Department of Experimental and Applied Psychology

^2^ The Netherlands Institute for the Study of Crime and Law Enforcement (NSCR)

^3^ Maastricht University

^4^ Kieskompas – Election Compass, Amsterdam, the Netherlands

^5^ The Pennsylvania State University, Department of Political Science

^6^ VU Amsterdam, Department of Communication Science and Political Science

**Analysis of attrition**

Here, we also analyze the effects of the predictor variables on attrition for the raw, unweighted data (note that the sample reported in the paper was weighted at T2, so the effects of attrition on the interpretation of the main results should be minimal). For this purpose, we conducted two logistic regressions. In both of them, we included the control variables (gender, age, education, and political orientation) in Step 1, either Covid-19 conspiracy beliefs or conspiracy mentality in Step 2, and participation as dependent variable (1 = only participated at T1, 2 = also participated at T2).

For *Covid-19 conspiracy beliefs*, Step 1 was significant, χ^2^(4) = 535.38, *p* < .001 (Nagelkerke *R*^2^ = .084). Results indicated that men were more likely than women to participate in both waves, *B* = -0.238, *SE* = .051, Wald = 22.02, *p* < .001; Exp(*B*) = 0.788, CI_95%_[0.714; 0.871]. Moreover, participation in both waves was predicted by both older age, *B* = 0.032, *SE* = .002, Wald = 425.29, *p* < .001; Exp(*B*) = 1.032, CI_95%_[1.029; 1.035], and higher education, *B* = 0.077, *SE* = .019, Wald = 15.87, *p* < .001; Exp(*B*) = 1.080, CI_95%_[1.040; 1.122]. The effect of political orientation was not significant, *p* = .277. Step 2 that added Covid-19 conspiracy beliefs to the model was significant, χ^2^(1) = 10.57, *p* = .001 (Nagelkerke *R*^2^ = .086). Covid-19 conspiracy beliefs at T1 predicted a decreased likelihood participating again at T2, *B* = -0.052, *SE* = .016, Wald = 10.61, *p* = .001; Exp(*B*) = 0.949, CI_95%_[0.920; 0.979].

For *conspiracy mentality,* Step 1 was similar as for Covid-19 conspiracy beliefs, albeit with slightly different statistics due to missing values, χ^2^(4) = 518.30, *p* < .001 (Nagelkerke *R*^2^ = .088). Gender, age, and education (but not political orientation) again predicted attrition. Step 2 that added conspiracy mentality to the model also was significant, χ^2^(1) = 5.89, *p* = .015 (Nagelkerke *R*^2^ = .088). Higher conspiracy mentality predicted a decreased likelihood of participating again at T2, although the effect size was small, *B* = -0.031, *SE* = .013, Wald = 5.89, *p* = .015; Exp(*B*) = 0.970, CI_95%_[0.946; 0.994].
